# Patterns of neurogenic lower urinary tract dysfunction management and associated factors among Chinese community-dwelling individuals with spinal cord injury

**DOI:** 10.1038/s41598-024-64081-w

**Published:** 2024-06-09

**Authors:** Mengyang Zhang, Ye Chen, Jiawei Liu, Can Luo, Zhong Chen, Tao Xu

**Affiliations:** 1 Department of Rehabilitation, Tongji Hospital Affiliated to Tongji Medical College of Huazhong University of Science and Technology, Wuhan, 430030 Hubei China; 2 Department of Nursing, Tongji Hospital Affiliated to Tongji Medical College of Huazhong University of Science and Technology, Wuhan, 430030 Hubei China; 3 Department of Urology, Tongji Hospital Affiliated to Tongji Medical College of Huazhong University of Science and Technology, Wuhan, 430030 Hubei China

**Keywords:** Spinal cord injury, Neurogenic lower urinary tract dysfunction, Latent class analysis, Intermittent catheterization, Community-dwelling, Epidemiology, Neurogenic bladder, Spinal cord diseases

## Abstract

To identify different patterns of neurogenic lower urinary tract dysfunction management among Chinese community-dwelling individuals with spinal cord injury and explore the factors associated with latent classes. This was a cross-sectional study conducted in communities throughout China Mainland. Participants were recruited through the China Association of Persons with Physical Disability and a total of 2582 participants was included in the analysis. The data were collected by a questionnaire consisting of socio-demographic factors, disease-related factors, and a list of 8 bladder management methods. Latent class analysis was used to identify different latent classes of neurogenic lower urinary tract dysfunction management. Then the multinomial logistic regression was applied to analyze the relationship between neurogenic lower urinary tract dysfunction management patterns and socio-demographic and disease-related factors. Neurogenic lower urinary tract dysfunction management pattern among community-dwelling individuals with spinal cord injury was divided into four latent classes: “urinal collecting apparatus dominated pattern” (40.3%), “bladder compression dominated pattern” (30.7%), “intermittent catheterization dominated pattern” (19.3%) and “urethral indwelling catheterization dominated pattern” (9.6%). Multinomial logistic regression analysis found that the employment status, residential region, nursing need, payment method for catheterization products, hand function, time since spinal cord injury, urinary incontinence and concerns about social interaction affected by urination problems were significantly associated with latent classes. Only 19.3% of people used the intermittent catheterization as their main neurogenic lower urinary tract dysfunction management method. More attention needs to be paid to the promotion of the standardization process of intermittent catheterization in community-dwelling individuals with spinal cord injury. The associated factors of the four classes can be used for tailored and targeted interventions to increase the use of intermittent catheterization.

## Introduction

Neurogenic lower urinary tract dysfunction (NLUTD) resulting from spinal cord injury (SCI) is a major medical and social problem worldwide. The incidence of SCI is 40–80/million population/year according to the report of the World Health Organization^1^, and there are about 120,000 new cases of SCI every year in Mainland China^2^. SCI leads to NLUTD in approximately 70.0–84.0% of patients^3^. In the European Association of Urology (EAU) guidelines, the primary aims for management of NLUTD are protection of the upper urinary tract (UUT), achievement (or maintenance) of urinary continence, restoration of lower urinary tract function, and improvement of the patient’s quality of life (QoL).

For community-dwelling individuals with NLUTD, the selectable methods for emptying the bladder and controlling incontinence mainly include: condom catheters, incontinence pads, triggered reflex voiding, bladder compression using Valsalva or Credé maneuvers, intermittent catheterization (IC), indwelling urethral catheterization (IUC), and suprapubic indwelling catheterization (SPIC)^4^. Among these methods, IC is currently accepted as the evidence-based best practice for NLUTD management, as long as dexterity or available caregiver support and body habitus allow access^5,6^. IC enables the patient to empty the bladder under low intravesical pressure condition to preserve UUT function; meanwhile, periodic bladder emptying can remove bacteria before their irreparable proliferation, reducing urologic complications such as urinary tract infection (UTI)^7^. Due to the overall economic level of the country and the uneven distribution of medical resources, the popularization of IC in China was limited; even now, some NLUTD patients know little about it or don’t accept it due to inconvenience and the difficulty of performing the technique^8,9^.

To further improve the QoL for community-dwelling individuals with SCI and reduce urinary complications, many clinical medical staffs are working on the popularization of IC. During NLUTD health education in subordinate hospitals and community populations, we discovered that many patients used combinations of several methods to empty the bladder or changed their primary NLUTD management method multiple times throughout the course of their condition. Similar findings have also been reported in other studies^10,11^. These findings indicate potential patterns in the use of different NLUTD management methods within the population. Given the heterogeneity in the NLUTD management methods used by community-dwelling individuals with SCI, it is important to identify whether latent subclasses exist that can help enhance targeted management for persons with specific patterns of NLUTD management.

Latent class analysis (LCA) is a data-driven statistical approach that estimates distinct subclasses based on the response pattern of an individual in the detection questions^12^. To our knowledge, no previous study has used a data-driven approach to explore the subclasses of NLUTD management methods within the population. Identifying subclasses of NLUTD management methods and exploring their prevalence and predictors will be crucial for practitioners to adopt a targeted approach to manage NLUTD in community-dwelling individuals with SCI. The objectives of this study are to identify distinct classes of NLUTD management methods among community-dwelling individuals with SCI, and to explore predictors of latent classes.

## Materials and methods

### Study design and participants

This cross-sectional study was conducted based on a convenience sample of community-dwelling people with SCI in four regions (eastern region, northeast region, central region and western region) in China, from August 3, 2020 to August 31, 2020. Participants were recruited through the China Association of Persons with Physical Disability, a nonprofit social organization formed voluntarily by people with physical disability. The questionnaire was collected via an online survey. A total of 3120 participants submitted questionnaires. Of which, 538 participants were excluded from the data analysis (52 questionnaires with missing values or duplicated, 486 participants could void spontaneously without assistance). Therefore, 2582 participants were included in the analysis.

### Measures

#### Socio-demographic factors

Socio-demographic characteristics, including age, gender, educational level, marital status, employment status, residential region, annual household income, affordable cost of catheterization, nursing needs, and methods of catheterization products payment were collected. The residential region was obtained by the response of “eastern China”, “northeastern China”, “central China” and “western China” based on the listing of the four major economic regions announced by the National Bureau of Statistics of China^13^. Information on nursing needs was obtained using the question: “Do you need a caregiver or family member to help you with your care?”, and the choice was classified as either “yes” or “no”.

#### Disease-related factors

The disease-related factors included 8 items such as time since injury, cause of SCI, SCI classification, hand function, urinary incontinence. Hand function was assessed through a three-point scale with the response categories of “normal”, “partial” and “incapable”. Information on the urinary incontinence was obtained using the question: “Do you have urinary incontinence?”.

### NLUTD management methods

The NLUTD management methods collected in this study were based on literature review and the EAU Guidelines on Neuro-Urology^4,14^. The following eight NLUTD management methods were assessed as follows: spontaneous voiding without assistance, condom catheter or incontinence pad, bladder compression, triggered reflex voiding, intermittent catheterization, indwelling urethral catheterization, suprapubic indwelling catheterization, and others (such as electro-stimulation, electro-magnetic ball valve, detrusor stimulation, sacral implants, conus implants, vesicostomy, etc.). For the eight items on NLUTD management, the response options were “yes” and “no”.

### Statistical analysis

The data were analyzed by the SPSS 24.0 and Mplus 8.0. LCA was conducted with the Mplus 8.0 to classify the participants based on their responses to the 8 NLUTD management method items. Models with different numbers of the latent classes were assessed, starting with one, until the best fitting model was obtained^12^. For model selection in LCA, lower Akaike information criterion (AIC), Bayesian information criterion (BIC) and aBIC (adjusted Bayesian information criterion) indicated better fitness^15^. Entropy represents the accuracy of model classification, with a value ≥ 0.800 indicating the classification accuracy exceeds 90.0%. Lo–Mendell–Rubin likelihood ratio test (LMR) and Bootstrap likelihood ratio test (BLRT) were performed to compare the fit of models that were nested in each other^16^. A significant *p*-value of LMR and BLRT implied that the *k* model was better than the *k*-1 model^16^.

After selecting the optimum number of latent classes, a multinominal logistic regression was conducted to examine the association of latent classes with the socio-demographic and disease-related factors. An exploration modeling strategy for variable selection was used in the multinominal logistic regression analysis. The level for statistical significance was set at a *p*-value < 0.050.

### Ethics approval and consent to participate

The study was approved by the Ethics Committee of Tongji Hospital, Tongji Medical College, and Huazhong University of Science and Technology, Wuhan, China (Approval No. TJ-IRB20210314). The survey was anonymous and voluntary. Before the participants gained access to the questionnaires, they were informed that their anonymity would be guaranteed and they had the right to withdraw at any time. Informed consent was obtained from all participants. The authors confirmed that all methods were performed in accordance with relevant guidelines and regulations.

### Preprint

A previous version^23^ of this manuscript was published as a preprint [10.21203/rs.3.rs-2263423/v1].

## Results

### Sample characteristics

The socio-demographic characteristics of participants are presented in Table 1. The mean age was 45.52 years (*SD* = 12.02). The majority of the participants in this study were men (71.0%) and had nursing need (81.4%). Of all participants, 1371 (53.1%) were married, 1110 (43.0%) were unemployed and 1444 (55.9%) received middle school or below education. Regarding the residential region of the participants, 31.4% were from Eastern China, 25.2% were from Northeastern China, 14.1% were from Central China, and 29.3% were from Western China. Regarding economic status, the majority of the participants (82.1%) had an annual household income of less than CNY 50,000 (6945 $) and 61.4% of the participants could afford less than CNY 300 (41 $) a month in catheterization. Moreover, 92.4% of the participants could not reimburse through medical insurance for the catheterization products.

**Table 1:** Socio-demographic characteristics of the participants (n = 2582).

Variables	N (%)	Mean ± *SD*
Age, years		45.52 ± 12.02
Gender		
Men	1833 (71.0)	
Women	749 (29.0)	
Educational level		
Middle school or below	1444 (55.9)	
High school	936 (36.3)	
College/university or above	202 (7.8)	
Marital status		
Single^a^	1211 (46.9)	
Married	1371 (53.1)	
Employment status		
Employed	790 (30.6)	
Retired	362 (14.0)	
Unemployed	1430 (55.4)	
Region		
Eastern China	812 (31.4)	
Northeastern China	650 (25.2)	
Central China	364 (14.1)	
Western China	756 (29.3)	
Annual household income		
< 50,000CNY (6945$)	2120 (82.1)	
50,000–100,000CNY (6945–13,891$)	356 (13.8)	
100,000–150,000CNY (13,891–20,837$)	61 (2.4)	
> 150,000CNY (20,837$)	45 (1.7)	
Affordable cost in catheterization (per month)		
< 300 CNY (41$)	1586 (61.4)	
300–600 CNY (41–83$)	490 (19.0)	
600–1000 CNY (83–139$)	262 (10.1)	
1000–1500 CNY (139–208$)	131 (5.1)	
> 1500 CNY (208$)	113 (4.4)	
Nursing need^b^		
Yes	2103 (81.4)	
No	479 (18.6)	
Methods of catheterization products payment		
Medical insurance	195 (7.6)	
Self-supporting	2387 (92.4)	

^a^Including single, widowed, divorced, and separated; ^b^Do you need a caregiver or family member to help you with your care? *SD* standard deviation.

### Latent class analysis

Model fit statistics for LCA models with the different number of classes are shown in Table 2. The model with one to five classes was compared according to fit statistics, and the four-class model was selected because it obtained the lowest AIC, BIC, and adjusted BIC value. In addition, the LMR and BLRT were conducted between the three- and four-class models with *p*-values of < 0.050, indicating that four classes model was better.

**Table 2:** Latent class model fit comparison (n = 2582).

Latent classes	AIC	BIC	adjusted BIC	entropy	LMR *p*-value	BLRT *p*-value
1	16,591.287	16,638.137	16,612.719	–	–	–
2	16,223.239	16,322.796	16,268.783	0.798	< 0.001	< 0.001
3	15,656.686	15,808.950	15,726.341	0.908	< 0.001	< 0.001
4	15,113.134	15,318.105	15,206.100	0.901	< 0.001	< 0.001
5	15,149.149	15,406.827	15,267.026	0.916	0.001	< 0.001
6	14,764.656	15,075.040	14,906.645	0.937	0.336	0.088

– Not applicable, *AIC* Akaike information criterion, *BIC* Bayesian information criterion, *LMR* Lo–Mendell–Rubin likelihood ratio test, *BLRT* bootstrap likelihood ratio test.

**Figure 1:**
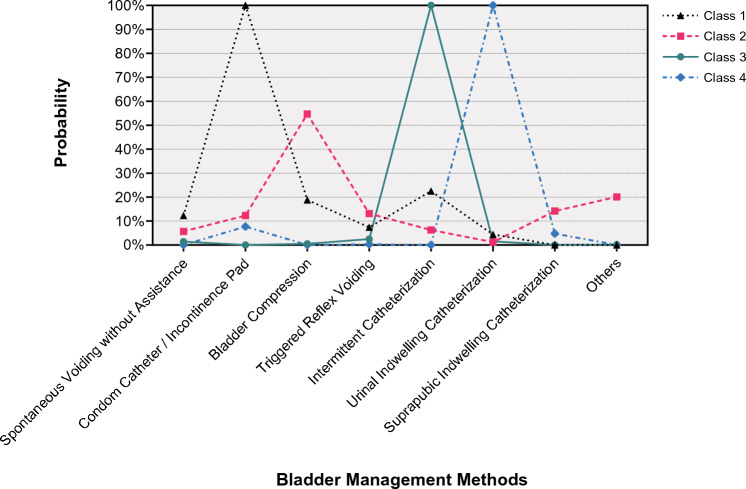
Probability of indicators of NLUTD management methods by latent class.

The quality of latent class classification for the four-class model was adequate (entropy = 0.901). Class 1 was the largest (n = 1041, 40.3%), followed by Class 2 (n = 793, 30.7%), Class 3 (n = 498, 19.3%), Class 4 (n = 250, 9.6%). Figure 1 showed the distribution of NLUTD management methods across each class. Classes were defined based on the item response probabilities conditional on class classification: Class 1 as “urinal collecting apparatus dominated pattern” because it was characterized by the high probability of using condom catheters/incontinence pads (100.0%) and low probability of using the seven other methods (0.0–22.5%); Class 2 as “bladder compression dominated pattern” because participants in this class had a high probability of using bladder compression (54.7%) and low probability of use of the seven other methods (1.2–20.1%); Class 3 as “IC dominated pattern” because participants assigned to this group had a high probability of using IC (100.0%), with a low probability of using in the seven other methods (0.0–2.5%); Class 4 as “IUC dominated pattern” because the high probability of using IUC (100.0%) were observed in these participants, with a low probability of using in the seven other methods (0.0–7.7%). In the raw data, the probability of using condom catheters/incontinence pads was 45.9%, bladder compression was 25.8%, IC was 29.2%, and IUC was 12.5%. The specific probability data of each class and raw data were shown in Supplementary material 1.

### Factors associated with latent classes

To explore the factors affecting latent classes, a univariate analysis was performed with socio-demographic factors and disease-related factors. The specific results are presented in Tables 3. The factors significantly associated with latent classes in univariate analysis were included in the multinomial logistic regression analysis. The multinomial logistic regression was fitted to four LCA-derived classes with the “IC dominated pattern” as reference. Compared with the IC dominated pattern class, members of all other classes were more likely to have longer time since injury and poorer hand function. Members of Class 1 and Class 4 were more likely to be unemployed with adjusted odd ratios (aOR) of 0.420 for Class 1 and 0.465 for Class 4 compared with the IC dominated pattern class. It was found that those in Class 1 and Class 2 were more likely to bear the cost of catheterization products themselves with aOR of 3.799 and 2.223, respectively. Members of Class 2 and Class 4 were less likely to report their social interaction affected by urination problems with aOR of 0.470 and 0.593 respectively compared with the IC dominated pattern class. Furthermore, living in northeastern China (aOR = 1.529) and having urinary incontinence (aOR = 2.282) were the variables that predicted the urinal collecting apparatus dominated pattern latent classes. Having no nursing need (aOR = 0.665) was the variable that predicted the bladder compression dominated pattern latent classes. Living in western China (aOR = 1.578) was the variable that predicted the IUC dominated pattern latent classes. Table 4 presented the results of the multinomial logistic regression analysis.

**Table 3:** Relationships between the socio-demographic factors, disease-related factors and latent classes (n = 2582).

Variables	Class 1 (n = 1041)	Class 2 (n = 793)	Class 3 (n = 498)	Class 4 (n = 250)	*F/χ*^2^	*p*-value
Age (years)	46.54 ± 12.36	44.33 ± 11.15	44.41 ± 12.27	47.28 ± 12.27	8.26	< 0.001
Gender					8.61	0.035
Men	707 (67.9)	580 (73.1)	359 (72.1)	187 (74.8)		
Women	334 (32.1)	213 (26.9)	139 (27.9)	63 (25.2)		
Educational level					26.78	< 0.001
Middle school or below	635 (61.0)	421 (53.1)	247 (49.6)	141 (56.4)		
High school	324 (31.1)	310 (39.1)	205 (41.2)	97 (38.8)		
College/University or above	82 (7.9)	62 (7.8)	46 (9.2)	12 (4.8)		
Marital status					9.91	0.019
Single^a^	500 (48.0)	391 (49.3)	203 (40.8)	117 (46.8)		
Married	541 (52.0)	402 (50.7)	295 (59.2)	133 (53.2)		
Employment status					63.15	< 0.001
Employed	333 (32.0)	244 (30.8)	146 (29.3)	67 (26.8)		
Retired	89 (8.5)	158 (19.9)	89 (17.9)	26 (10.4)		
Unemployed	619 (59.5)	391 (49.3)	263 (52.8)	157 (62.8)		
Region					23.27	0.006
Eastern China	306 (29.4)	263 (33.2)	172 (34.5)	71 (28.4)		
Northeastern China	307 (29.5)	180 (22.7)	98 (19.7)	65 (26.0)		
Central China	138 (13.3)	117 (14.8)	76 (15.3)	33 (13.2)		
Western China	290 (27.9)	233 (29.4)	152 (30.5)	81 (32.4)		
Annual household income					16.43	0.053
< 50,000 CNY (6945$)	866 (83.2)	649 (81.8)	390 (78.3)	215 (86.0)		
50,000–100,000 CNY (6945–13,891$)	141 (13.5)	108 (13.6)	79 (15.9)	28 (11.2)		
100,000–150,000 CNY (13,891–20,837$)	15 (1.4)	24 (3.0)	16 (3.2)	6 (2.4)		
> 150,000 CNY (20,837$)	19 (1.8)	12 (1.5)	13 (2.6)	1 (0.4)		
Affordable cost of catheterization (per month)					23.13	0.027
< 300 CNY (41$)	629 (60.4)	512 (64.6)	287 (57.6)	158 (63.2)		
300–600 CNY (41–83$)	213 (20.5)	132 (16.6)	97 (19.5)	48 (19.2)		
600–1000 CNY (83–139$)	106 (10.2)	74 (9.3)	58 (11.6)	24 (9.6)		
1000–1500 CNY (139–208$)	53 (5.1)	29 (3.7)	38 (7.6)	11 (4.4)		
> 1500 CNY (208$)	40 (3.8)	46 (5.8)	18 (3.6)	9 (3.6)		
Nursing need^b^					32.87	< 0.001
Yes	875 (84.1)	597 (75.3)	410 (82.3)	221 (88.4)		
No	166 (15.9)	196 (24.7)	88 (17.7)	29 (11.6)		
Methods of catheterization products payment					67.98	< 0.001
Medical insurance	42 (4.0)	51 (6.4)	58 (11.6)	44 (17.6)		
Self-supporting	999 (96.0)	742 (93.6)	440 (88.4)	206 (82.4)		
Time since injury (years)	12.1 (6.4, 21.5)	14.3 (7.6, 23.0)	7.1 (2.9, 17.0)	11.4 (6.0, 19.9)	103.51	< 0.001
Cause of spinal cord injury					3.25	0.355
Traumatic	876 (84.1)	684 (86.3)	415 (83.3)	217 (86.8)		
Non-traumatic	165 (15.9)	109 (13.7)	83 (16.7)	33 (13.2)		
SCI classification					38.75	< 0.001
Paraplegia	852 (81.8)	637 (80.3)	444 (89.2)	214 (85.6)		
Tetraplegia	147 (14.1)	94 (11.9)	31 (6.2)	30 (12.0)		
Unknown	42 (4.0)	62 (7.8)	23 (4.6)	6 (2.4)		
Hand function					52.11	< 0.001
Normal	592 (56.9)	515 (64.9)	350 (70.3)	125 (50.0)		
Partial	224 (21.5)	142 (17.9)	93 (18.7)	57 (22.8)		
Incapable	225 (21.6)	136 (17.2)	55 (11.0)	68 (27.2)		
Urinary incontinence					32.72	< 0.001
No	16 (1.5)	49 (6.2)	17 (3.4)	4 (1.6)		
Yes	1025 (98.5)	744 (93.8)	481 (96.6)	246 (98.4)		
Social interaction affected by urination problems					20.50	< 0.001
No	105 (10.1)	122 (15.4)	39 (7.8)	30 (12.0)		
Yes	936 (89.9)	671 (84.6)	459 (92.2)	220 (88.0)		
Do you worried about urethral injury caused by urethral catheterization?					19.00	0.020
No	70 (6.7)	77 (9.7)	38 (7.6)	11 (4.4)		
Yes	971 (93.3)	716 (90.3)	460 (92.4)	239 (95.6)		
Do you worried about the pain caused by urethral catheterization?					7.46	0.059
No	170 (16.3)	141 (17.8)	101 (20.3)	32 (12.8)		
Yes	871 (83.7)	652 (82.2)	397 (79.7)	218 (87.2)		

Class 1 = urinal collecting apparatus dominated pattern; Class 2 = bladder compression dominated pattern; Class 3 = IC dominated pattern; Class 4 = IUC dominated pattern; ^a^Including single, widowed, divorced, and separated; ^b^Do you need a caregiver or family member to help you with your care? Group comparisons were performed using analysis of variance (ANOVA) and Kruskal–Wallis test for continuous variables, or Fisher’s exact test and χ^2^ test for categorical variables; Values were presented as Mean ± *SD*, median (IQR), number (%).

**Table 4:** Multinomial logistic regression analysis of NLUTD management patterns (reference = class 3).

Variable	*aOR*	95% *CI*
Class 1: urinal collecting apparatus dominated pattern		
Socio-demographic factors		
Age (years)	1.001	(0.989, 1.014)
Gender (vs. men)		
Women	1.241	(0.966, 1.595)
Educational level (vs. college/university or above)		
Middle school or below	1.345	(0.887, 2.040)
High school	0.880	(0.578, 1.341)
Marital status (vs. single)		
Married	0.810	(0.601, 1.020)
Employment status (vs. unemployed)		
Employed	1.270	(0.981, 1.644)
Retired	0.420***	(0.299, 0.590)
Region (vs. Eastern China)		
Northeastern China	1.529**	(1.110, 2.106)
Central China	1.095	(0.767, 1.565)
Western China	1.148	(0.859, 1.534)
Affordable cost of catheterization (per month) (vs. < 300 CNY (41$))		
300–600 CNY (41–83$)	1.054	(0.579, 1.918)
600–1000 CNY (83–139$)	0.716	(0.449, 1.141)
1000–1500 CNY (139–208$)	0.843	(0.583, 1.219)
> 1500 CNY (208$)	1.104	(0.825, 1.478)
Nursing need (vs. no)		
Yes	0.878	(0.642, 1.201)
Methods of catheterization products payment (vs. medical insurance)		
Self-supporting	3.799***	(2.460, 5.867)
Disease-related factors		
Time since injury (years)	1.030***	(1.018, 1.043)
SCI classification (vs. tetraplegia)		
Paraplegia	0.723	(0.430, 1.215)
Unknown	0.606	(0.291, 1.263)
Hand function (vs. normal)		
Partial	1.441*	(1.074, 1.934)
Incapable	2.156***	(1.411, 3.295)
Urinary incontinence (vs. no)		
Yes	2.282*	(1.050, 4.957)
Social interaction affected by urination problems (vs. no)		
Yes	0.752	(0.507, 1.115)
Worried about urethral injury caused by urethral catheterization? (vs. no)		
Yes	0.893	(0.567, 1.404)
Class 2: bladder compression dominated pattern		
Socio-demographic factors		
Age (years)	0.986	(0.969, 1.004)
Gender (vs. men)		
Women	0.849	(0.649, 1.111)
Educational level (vs. college/university or above)		
Middle school or below	1.184	(0.761, 1.842)
High school	1.060	(0.681, 1.651)
Marital status (vs. single)		
Married	0.886	(0.682, 1.150)
Employment status (vs. unemployed)		
Employed	0.996	(0.801, 1.210)
Retired	1.056	(0.910, 1.421)
Region (vs. Eastern China)		
Northeastern China	1.160	(0.827, 1.626)
Central China	1.152	(0.797, 1.666)
Western China	1.122	(0.829, 1.517)
Affordable cost of catheterization (per month) (vs. < 300 CNY (41$))		
300–600 CNY (41–83$)	1.462	(0.790, 2.574)
600–1000 CNY (83–139$)	0.684	(0.285, 1.220)
1000–1500 CNY (139–208$)	0.763	(0.516, 1.127)
> 1500 CNY (208$)	0.857	(0.627, 1.172)
Nursing need (vs. no)		
Yes	0.665*	(0.486, 0.910)
Methods of catheterization products payment (vs. medical insurance)		
Self-supporting	2.223***	(1.462, 3.381)
Disease-related factors		
Time since injury (years)	1.048***	(1.035, 1.061)
SCI classification (vs. tetraplegia)		
Paraplegia	0.649	(0.376, 1.120)
Unknown	0.936	(0.448, 1.956)
Hand function (vs. normal)		
Partial	1.237	(0.904, 1.693)
Incapable	1.697*	(1.085, 2.654)
Urinary incontinence (vs. no)		
Yes	0.699	(0.363, 1.349)
Social interaction affected by urination problems (vs. no)		
Yes	0.470***	(0.318, 0.693)
Worried about urethral injury caused by urethral catheterization? (vs. no)		
Yes	0.929	(0.586, 1.472)
Class 4: IUC dominated pattern		
Socio-demographic factors		
Age (years)	1.014	(0.996, 1.032)
Gender (vs. men)		
Women	1.021	(0.711, 1.464)
Educational level (vs. college/university or above)		
Middle school or below	1.774	(0.885, 3.557)
High school	1.613	(0.802, 3.243)
Marital status (vs. single)		
Married	0.723	(0.510, 1.025)
Employment status (vs. unemployed)		
Employed	0.907	(0.621, 1.326)
Retired	0.465**	(0.283, 0.764)
Region (vs. Eastern China)		
Northeastern China	1.141	(0.726, 1.793)
Central China	1.162	(0.689, 1.959)
Western China	1.578*	(1.041, 2.391)
Affordable cost of catheterization (per month) (vs. < 300 CNY (41$))		
300–600 CNY (41–83$)	0.887	(0.382, 2.058)
600–1000 CNY (83–139$)	0.641	(0.312, 1.316)
1000–1500 CNY (139–208$)	0.763	(0.447, 1.304)
> 1500 CNY (208$)	0.898	(0.593, 1.360)
Nursing need (vs. no)		
Yes	1.037	(0.634, 1.696)
Methods of catheterization products payment (vs. medical insurance)		
Self-supporting	0.706	(0.446, 1.116)
Disease-related factors		
Time since injury (years)	1.018*	(1.001, 1.034)
SCI classification (vs. tetraplegia)		
Paraplegia	1.226	(0.636, 2.360)
Unknown	0.738	(0.242, 2.249)
Hand function (vs. normal)		
Partial	1.768**	(1.178, 2.653)
Incapable	3.628***	(2.159, 6.096)
Urinary incontinence (vs. no)		
Yes	1.769	(0.540, 5.791)
Social interaction affected by urination problems (vs. no)		
Yes	0.593*	(0.355, 0.992)
Worried about urethral injury caused by urethral catheterization? (vs. no)		
Yes	1.439	(0.672, 3.082)

*p < 0.05, **p < 0.01, ***p < 0.001. The reference category was Class 3 (IC dominated pattern); *aOR* adjusted Odds Ratio, *CI* Confidence Intervals.

## Discussion

In the present study, we leveraged LCA to identify the distinct patterns of NLUTD management among individuals with SCI living in the community, and explored the association of latent classes with socio-demographic and disease-related factors. Our findings indicate that almost 82.8% (2582/3120) of community-dwelling individuals with SCI could not fully control their urination and required supplemental methods to manage NLUTD. This prevalence is consistent with previous research on NLUTD in SCI patients^3^. Given the high prevalence of NLUTD in this population, it is crucial for rehabilitation and urology professionals to provide more attention and interventions.

The LCA results identified a 4-class model. 40.3% of our participants (Class 1) tended to use the urinal collecting apparatus (condom catheter or incontinence pad) for NLUTD management. The multinomial logistic regression showed that urinary incontinence was significantly associated with Class 1. The finding was similar to the study in Denmark, which found the most common combination of the bladder-emptying method was IC and the use of urinal collecting apparatus due to the incontinence not being easy to overcome^10^. However, the urinal collecting apparatus dominated pattern is too passive which means that patients rely primarily on incontinence to urinate and may bring high risk of UTI and high intravesical pressure^4^. Persons suffered from urinary incontinence should adjust their medication to control DO, or change the IC protocol to accommodate the increased urine leaks under medical guidance. The urinal collecting apparatus should be utilized as an auxiliary method to combat incontinence rather than being the main approach for NLUTD management.

Class 2 represented 30.7% of the sample consisted of individuals who relied on bladder compression such as Credé or Valsalva maneuver to void. This approach may lead to increased intravesical pressure and bladder outlet resistance due to reflex sphincter contraction^4,17^, which might cause urological complications such as structural bladder damage, vesicoureteral reflux (VUR), hydronephrosis, and renal insufficiency^4,18^. Another manually assisted voiding method, triggered reflex voiding, which is not always achievable and the maneuvers are unique to each person and too difficult to master^19^, showed a low usage rate among participants (13.1%) and was highest in Class 2. This indicates that when individuals are unable to successfully urinate using the triggered reflex, they resort to bladder compression techniques. It is important to note that these manually assisted voiding methods should only be attempted in patients with confirmed safe urodynamic parameters. Due to the associated complications with UUT, these methods are no longer routinely recommended.

Only 19.3% of our participants belonged to Class 3 (IC dominated pattern), who tended to use IC for NLUTD management. As the mainstream NLUTD management method worldwide, IC has a low complication rate and improves continence, leading to greater community participation and decreasing home confinement^19^. However, our results suggest that the penetration rate of IC remains low in China. Class 4 (IUC dominated pattern) represented 9.6% of our participants. IUC is generally reserved for patients who are unable or unwilling to perform IC and have contraindications to other options like SPIC^19^. However, in general, IUC should only be used as a last resort due to its high complication rate, including urethral erosion, fistula, epididymitis, and periurethral abscess^19^.

It is well known that IC is the safest NLUTD management method for patients with SCI in terms of urological complications^4^. The results of LCA indicated that the majority of community-dwelling individuals with NLUTD after SCI were still using non-recommended NLUTD management methods. Understanding the differences between Class 3 and the other classes will aid in promoting the adoption of IC and reducing the risk of urinary complications among community-dwelling individuals with SCI.

Multinomial logistic regression analysis revealed that impaired hand function and longer duration of SCI were associated with Classes 1, 2, and 4. Apparently, impaired hand function will make it more difficult for persons to self-catheter. For these people, SPIC may be more recommended than IUC, bladder compression, and urinal collecting apparatus in the absence of contraindications because its long-term outcomes are comparable with IC^20^. Regarding the duration of SCI, due to the late promotion of IC in China, people with a long course of SCI have less access to the IC. Therefore, the standardization process for IC should not be limited to hospitals but extended to community settings.

Unemployment was associated with Classes 1 and 4 compared to Class 3. Compared to urinal collecting apparatus and IUC, IC is more expensive due to the cost of consumed catheters^6,21^. In Class 1 and Class 2, self-supporting payment for catheterization products was a negative predictor of IC usage. Although some catheters are reused by patients after sterilization and lubrication, IC catheters still impose a significant financial burden on patients^7^. This suggests that reimbursements for intermittently used catheters could promote the use of IC by individuals with SCI. Despite potential burdens on medical insurance funding, IC remains highly cost-effective as it reduces long-term complications and related healthcare costs^6^. In Class 2 and Class 4, people would tend to use IC if the urination problems affected their social interaction. As previously mentioned, IC enables greater community participation, making it the best choice for individuals who value social interactions^19^. The lack of such differences in Class 1 may be attributed to the fact that the urinal collecting apparatus also allows for social continence. Combining urinal collecting apparatus with IC may be a viable solution for individuals who prioritize social interaction but also experience incontinence^10^.

In Class 2, individuals requiring nursing assistance showed a preference for IC over bladder compression techniques. This may be because people can seek assistance from paramedics with IC in the presence of nursing personnel. Living in northeast China or western China was associated with Class 1 or Class 4, respectively. Western China's socio-economic development lags behind that of eastern China^22^. Inadequate healthcare spending and material scarcity may explain that the people who lived in western China tended to use IUC compared with IC. However, these factors may not completely account for the increased use of urinal collecting apparatus in northeast China. Other regional differences, such as medical insurance policies, may contribute to this discrepancy.

There are some limitations in this study. First, our study had a cross-sectional design that prevented us from identifying causal relationships between variables. And the bladder emptying method could be changed over time according to the health condition or patients’/caregivers’ preference. In future research, longitudinal data should be collected to determine the causal relationships between these variables and to determine the trajectory of change in bladder management patterns. Second, due to the limited resources and manpower, we had not yet collected comprehensive data on the disease-related factors, such as neurogenic bladder symptom score and catheter-associated UTIs. Future studies should aim to collect this data in order to explore the best management strategies for NLUTD in community-dwelling individuals with SCI. Third, we did not collect the data on the classification of NLUTD and medications. Because the questionnaires in our study were self-reported, considering that the classification of NLUTD is highly specialized, it may be difficult for patients to accurately respond to such specialized data. And the participants in our study were spinal cord injury patients who often take multiple medications. Therefore, these data were not collected in order to ensure the accuracy of the results. Fourth, our study used a convenient sampling method, so generalizations about conclusions need to be made with caution.

In conclusion, the present study identified four classes of NLUTD management patterns in community-dwelling individuals with SCI. The most prevalent pattern was the use of urinal collecting apparatus, while only 19.3% of participants preferred IC. Factors such as impaired hand function, longer time since SCI, and availability of self-supporting catheterization products influenced individuals’ decision to use IC as their primary NLUTD management method. Those who were bothered by urinary incontinence tended to use urinal collecting apparatus, while individuals who faced social challenges related to urination and retired individuals were more inclined to use IC. These findings can guide tailored interventions aimed at improving NLUTD management in community-dwelling individuals with SCI.

### Supplementary Information

Supplementary Information.

## Data Availability

The data that support the findings of this study are not publicly available due to them containing information that could compromise research participant consent but are available from the corresponding author on reasonable request.

## Abbreviations

aBIC: Adjusted Bayesian information criterion
AIC: Akaike information criterion
ANOVA: Analysis of variance
aOR: Adjusted odds ratio
BIC: Bayesian information criterion
BLRT: Bootstrap likelihood ratio test
CI: Confidence interval
DO: Detrusor overactivity
EAU: European Association of Urology
IC: Intermittent catheterization
LCA: Latent class analysis
LMR: Lo–Mendell–Rubin likelihood ratio test
NLUTD: Neurogenic lower urinary tract dysfunction
QoL: Quality of life
SCI: Spinal cord injury
SD: Standard deviation
SPIC: Suprapubic indwelling catheterization
IUC: Indwelling urethral catheterization
UTI: Urinary tract infection
UUT: Upper urinary tract
VUR: Vesicoureteral reflux
