# Supplementary material for: Patterns of neurogenic lower urinary tract dysfunction management and associated factors among Chinese community-dwelling individuals with spinal cord injury
Source: Sci Rep. 2024 Jun 9;14:13224. doi: 10.1038/s41598-024-64081-w (PMC11162410; doi:10.1038/s41598-024-64081-w)
Supplement: Supplementary file 1 — Supplementary Information. [file 41598_2024_64081_MOESM1_ESM.pdf]

Distribution of individual bladder management methods in each class and raw data (probability data)

bladder management method	Class 1	Class 2	Class 3	Class 4	raw data
**Spontaneous Voiding without Assistance**	0.123	0.057	0.014	0	0.067
**Condom Catheter/Incontinence Pad**	1	0.123	0	0.077	0.459
**Bladder Compression**	0.188	0.547	0.005	0	0.258
**Triggered Reflex Voiding**	0.074	0.131	0.025	0.003	0.077
**Intermittent Catheterization**	0.225	0.063	1	0	0.292
**Indwelling Urethral Catheterization**	0.044	0.012	0.015	1	0.125
**Suprapubic Indwelling Catheterization**	0	0.142	0	0.048	0.053
**Others**	0	0.201	0.002	0	0.069

Note: The sum of the probabilities of the raw data exceeding 100% is due to the fact that some patients used multiple methods to empty their bladder at the same time.
